# Q-PCR Based Culture-Independent Enumeration and Detection of *Enterobacter*: An Emerging Environmental Human Pathogen in Riverine Systems and Potable Water

**DOI:** 10.3389/fmicb.2016.00172

**Published:** 2016-02-17

**Authors:** Chandra B. Patel, Rishi Shanker, Vijai K. Gupta, Ram S. Upadhyay

**Affiliations:** ^1^Environmental Toxicology Group, CSIR-Indian Institute of Toxicology ResearchLucknow, India; ^2^Department of Botany, Banaras Hindu UniversityVaranasi, India; ^3^Institute of Life Sciences, School of Life Science and Technology, Ahmedabad UniversityAhmedabad, India; ^4^Molecular Glycobiotechnology Group, Discipline of Biochemistry, School of Natural Sciences, National University of Ireland GalwayGalway, Ireland

**Keywords:** fecal coliforms, microbial contamination, real-time PCR, riverine system, quantitative enumeration, *Enterobacter*

## Abstract

The availability of safe and pristine water is a global challenge when large numbers of natural and anthropogenic water resources are being depleted with faster rate. The remaining water resources are severely contaminated with various kinds of contaminants including microorganisms. *Enterobacter* is one of the fecal coliform bacteria of family Enterobacteriaceae. *Enterobacter* was earlier used as an indicator bacterium along with other fecal Coliforms namely *Escherichia coli*, *Citrobacter*, and *Klebsiella*, but it is now known to cause various diseases in human beings. In this study, we have collected 55 samples from potable water and riverine system and proved their presence using their conserved sequences of 16S rRNA and 23S rRNA genes with the help of SYBR green real-time PCR, which showed very high specificity for the detection of *Enterobacter.* The *Enterobacter* counts in potable water were found to 1290 ± 32.89 to 1460 ± 39.42 cfu/100 ml. The *Enterobacter* levels in surface water were 1.76 × 10^4^ ± 492, 1.33 × 10^4^ ± 334, 1.15 × 10^4^ ± 308, 2.56 × 10^4^ ± 802, 2.89 × 10^4^ ± 962, 8.16 × 10^4^ ± 3443 cfu/100 ml; the levels of *Enterobacter* contamination associated with hydrophytes were 4.80 × 10^4^ ± 1804, 3.48 × 10^4^ ± 856, 8.50 × 10^4^ ± 2074, 8.09 × 10^4^ ± 1724, 6.30 × 10^4^ ± 1738, 3.68 × 10^4^ ± 949 cfu/10 g and the *Enterobacter* counts in sediments of the river, were 2.36 × 10^4^ ± 703, 1.98 × 10^4^ ± 530, 9.92 × 10^4^ ± 3839, 6.80 × 10^4^ ± 2230, 8.76 × 10^4^ ± 3066 and 2.34 × 10^4^ ± 732 cfu/10 g at the sampling Site #1, Site #2, Site #3, Site #4, Site #5, and Site #6, respectively. The assay could be used for the regular monitoring of potable water and other water reservoirs to check waterborne outbreaks.

## Introduction

The change in the global environment has led to the inappropriate distribution of microbial species, which resulted into emergence and re-emergence of microbial pathogens. The maximum effect of ecological imbalance is observed in the aquatic environment. In India and other developing countries the availability of safe and pristine potable water is a challenging task. Surface and potable waters are heavily contaminated with microorganisms leading to various waterborne diseases and outbreaks. Hence, the microbiological examination of water is used worldwide to monitor and control the quality and safety of various types of water. Many potential pathogens remain associated with water and it is thus impractical to screen samples for all possible pathogens. Hence some indicator microorganisms are required to trace the presence of possible pathogens in the aquatic environment. The guidelines and regulations for safe recreational and potable waters require determination of the absence of ‘indicator’ microorganisms. The most widely used indicator microbial systems are ‘coliform bacteria.’ Coliform bacteria normally exist in the intestinal tract of warm-blooded animals and humans, and are discharged through fecal matter. In addition, most coliform bacteria can also exist widely in the natural environment, including soil, surface water, and to some extent in groundwater (Francy et al., 2000). Fecal coliform bacteria are the most common pollutant in rivers and streams (Hill et al., 2006). Non-pathogenic coliforms can survive in water in adverse conditions as compared to other pathogenic bacteria. Total coliforms belong to the family Enterobacteriaceae and have been defined as facultative anaerobic, Gram-negative, non-spore-forming, rod-shaped bacteria that ferment lactose with gas and acid formation within 48 h at 35°C (American Public Health Association [APHA], 1998). Fecal coliforms are thermo-tolerant with same fermentation properties as total coliforms but at 44 ± 0.5°C. There are four genera of fecal coliforms namely *Escherichia, Klebsiella, Enterobacter*, and *Citrobacter*.

*Enterobacter* is a Gram-negative, rod-shaped bacterium belonging to the family Enterobacteriaceae. Hormaeche and Edwards (1960) first described the genus *Enterobacter* and under this genus, 14 species are included (Abbott, 2003). *Enterobacter* is an opportunistic pathogen that has been associated with food-borne illness (Mullane et al., 2006, 2007), septicemia and meningitis in preterm and full-term infants (Lai, 2001; Gurtler and Beuchat, 2005) and necrotizing enterocolitis in neonates (van Acker et al., 2001). The yellow-pigmented coliform was the causative bacterium in a case of septicemia in an infant (Pangalos, 1929). On the basis of the abundance of genus *Enterobacter, Enterobacter cloacae* has been the most frequently isolated species in humans, followed by *E. aerogenes* and *E. agglomerans. E. sakazakii* and *E. gergoviae* are uncommon human pathogens (Ristuccia and Cunha, 1985). Therefore, in this study, we have detected the *Enterobacter* sp. in the environmental samples such as potable water, surface water, and sediments of the river by targeting 16S rRNA and 23S rRNA genes by SYBR Green based quantitative PCR (qPCR).

River Gomti is the lifeline of Lucknow city (population more than 5 million, according to census-2011), the state capital of Uttar Pradesh, and it supplies about half of the total domestic water demand (about 550 mLd) of the town (Singh et al., 2004). However, river Gomti is identified as one of the most polluted rivers in India which contain huge amounts of organic and inorganic wastes leading to eutrophication of the river water (Singh et al., 2004) and hence bacteria flourish. Depending on the origin the sources of pollution can be categorized into two main categories that is a point source of pollution and non-point sources of pollution. The known sources of pollution are called as a point source of pollution for example, household wastes, municipal wastes, industrial eﬄuents, etc. The unknown and unidentified sources of pollution are called as non-point sources of pollution for example agricultural runoff, storm water, wildlife animal wastes, etc.

Analysis of fecal-indicator bacteria by most probable number (MPN) procedures provides an indication of fecal pollution in water (World Health Organization [WHO], 2002). However, 48 h is required to confirm fecal pollution in a sample. However, this provides no insight about the coliform bacteria (Ram et al., 2011).

## Materials and Methods

### Designing of Primers Specific to 16S rRNA and 23S rRNA

The complete coding sequences of 23S rRNA gene (accession numbers: DQ869858, DQ869859, DQ869860, and DQ869861) and 16S rRNA gene (accession numbers: AB274275, AB274276, AB274277, AB274278, AB274279, AB274280, AB274282, AB274283, AB274284, and AB274285) were retrieved from NCBI GenBank database^1^ to design a set of primers for specific detection of *Enterobacter* in environmental samples.

The alignment of the sequences retrieved for 23S rRNA and 16S rRNA genes were created separately using ClustalW program^2^ to determine the conserved sequences. The primers for 23S rRNA and 16S rRNA genes were designed in highly conserved region of the genome using Beacon Designer 5.0, Premier Biosoft International (**Table 1**). The specificity of primers was determined against known microbial genomes and sequences by BLAST (Basic Local Alignment Search Tool) program^3^ to ensure no homology was observed with known gene sequences of other waterborne pathogens. All the primers used in this study were synthesized from Metabion (Gmbh, Germany). Although, in blast search, it might have shown homology with *Klebsiella*, but before the use of these primers to detect *Enterobacter* from the environmental samples, the DNA was isolated from other Enterobacteria like *Escherichia coli, Klebsiella, Citrobacter* and were spiked with sterile water and used as DNA template followed by execution by PCR, which produced only specific product with *Enterobacter* species (**Figure 6**).

**Table 1:** Sequence of primers were used to detect *Enterobacter* in environmental samples.

Name of genes	Primers Sequences (5′-3′)	Length (nt)	Tm (°C)	% GC	Product Length (bp)
	F: Forward				
	R: Reverse				
23S rRNA	F: AGTGGAACGGTCTGGAAAGG	20	56.5	55	154
	R: TCGGTCAGTCAGGAGTATTTAGC	23	57.3	47.8	
16S rRNA	F: ATCAGATGTGCCCAG ATGG	19	53.5	52.6	110
	R: CCGTGTCTCAGTTCCAGTG	19	54.6	57.9	

### Generation of Standard Curve for Quantitative Enumeration of *Enterobacter* sp.

For preparation of the standard curve, the reference strain (*Enterobacter* MTCC 659 exhibiting 16S rRNA and 23S rRNA genes procured from the Microbial Type Culture Collection at Institute of Microbial Technology (IMTECH), Chandigarh, India) was grown in triplicate in 1 ml Luria-Bertani (LB) broth for 12 h at 37 ± 1°C to approximately optical density of 0.8 at 600 nm. The number of colony forming units (CFU/ml) of cell suspension was determined by plating 100 μl of the threefold diluted culture onto five Luria-Bertani agar plates. The average number of CFU from the five plates was used to calculate the concentration (CFU/ml) of cells in the culture. Further, triplicate cultures of a reference strain (optical density 0.8 at 600 NM) were serially diluted tenfold to yield 10^7^ down to 1 CFU/ml in phosphate-buffered saline as estimated by the standard plate count method. DNA template was prepared from each dilution as per Jyoti et al. (2010) and qPCR assays for both the genes were performed using an iCycler (BIO-RAD, USA) real-time PCR instrument and Quantifast SYBR Green PCR kit (Qiagen, Germany). Briefly, the reaction mixture contained 0.2 μM of the forward and reverse oligo nucleotide primer (1 μl each) for 16S/23S rRNA gene, 12.5 μl of Quantifast SYBR Green PCR kit (Qiagen, Germany), deionized water (5.5 μl nuclease free water provided by Qiagen, Germany) and 5 μl DNA template (corresponding to 10^6^-1 CFU/PCR) in a final volume of 25 μl. In negative controls 5 μl sterilized Milli-Q water used as a template. The PCR amplification protocol for the assay for both the genes consisted of an initial denaturation of 5 min at 95°C followed by 45 cycles of three steps consisting of 10 s at 95°C, 20 s at 55.8°C, and 20 s at 72°C. The fluorescence signals were measured at the end of each extension step. At the completion of PCR amplification, melting temperature (Tm) analysis of the products was performed by reducing the temperature to 65°C and then heating to 95°C at a rate of 0.2°C per second (**Figure 3**). Fluorescence was monitored continuously to confirm amplification specificity. The Tm peaks were calculated based on the initial fluorescence curve (F/T) by plotting the negative derivative of fluorescence over temperature versus temperature (–dF/dT versus T). The qPCR assays to generate standard curves for both the genes were repeated thrice and average PCR efficiencies and *R*^2^ values were calculated to check the reproducibility of the assays.

### Quantitative Enumeration of *Enterobacter* in Potable Water

**FIGURE 1:**
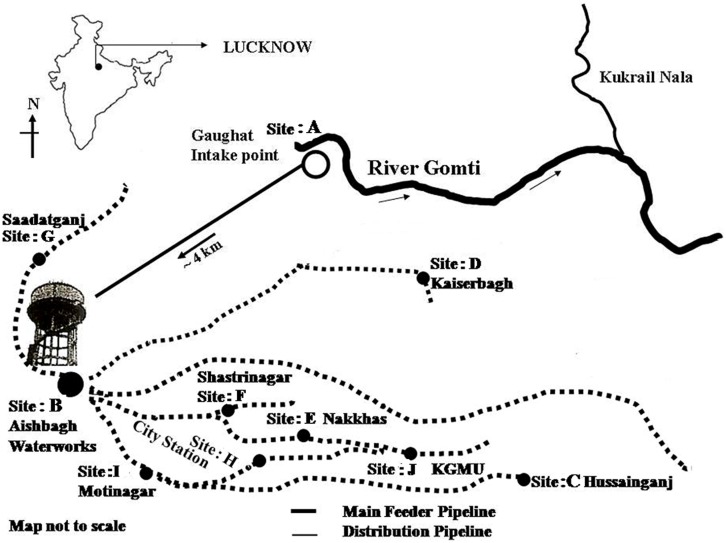
**Sampling sites of potable water in Lucknow City**.

For potable water supply in Lucknow (a major city of northern India) water is pumped from the river Gomti at Gaughat, which is outside the city, and is sent through a pipeline to Lucknow Jal Sansthan, Aishbagh, 4 km away (**Figure 1**) (Patel et al., 2011), where the water is purified by alum treatment, filtration, and chlorination before being released into the drinking water supply (Clever et al., 2000; Shrivastava et al., 2004; Ram et al., 2008). To test the possibility of the contamination of potable waters by *Enterobacter* due to defective water distribution systems and insufficient treatment during production, we collected 1 l water samples in triplicate for culture free quantitative enumeration at ten sampling sites: site A: Gaughat (raw water intake point in the river Gomti), site B: Aishbagh Waterworks (before water enters the distribution system); site C: Hussainganj; site D: Kaiserbagh (water-distribution pipeline that neither percolated nor ran along open drainage); site E: Nakkhas; site F: Shashtri Nagar; site G: Saadatganj; site H: City Station; site I: Moti Nagar (pipeline that percolated and ran along open drainage); and site: J, KGMU (**Figure 1**). All the potable water samples were collected in sterile glass bottles on the same day in densely populated areas across the urban boundaries of Lucknow city. The samples were stored on ice, and transported to the laboratory for analyses within 6 h (American Public Health Association [APHA], 1998 ^4^). The sites were numbered in order of the sample collection. Bacteriological quality of the potable water samples collected in this study was assayed to ascertain *Enterobacter* contamination. Total coliform and fecal coliform levels in potable water collected from each site were determined by the MPN method as per American Public Health Association [APHA], 1998). Further, multigenomic DNA from water (500 μL) was extracted by boiling lysis method as per the procedure described by Jyoti et al. (2010) and quantification of *Enterobacter* based on 16S rRNA and 23S rRNA genes was done by tenfold standard curve was generated by diluting the culture of *Enterobacter* MTCC 659.

### Quantitative Enumeration of *Enterobacter* in Riverine-Systems

#### Sampling of Surface Water from Riverine-Systems and DNA Isolation

**FIGURE 2:**
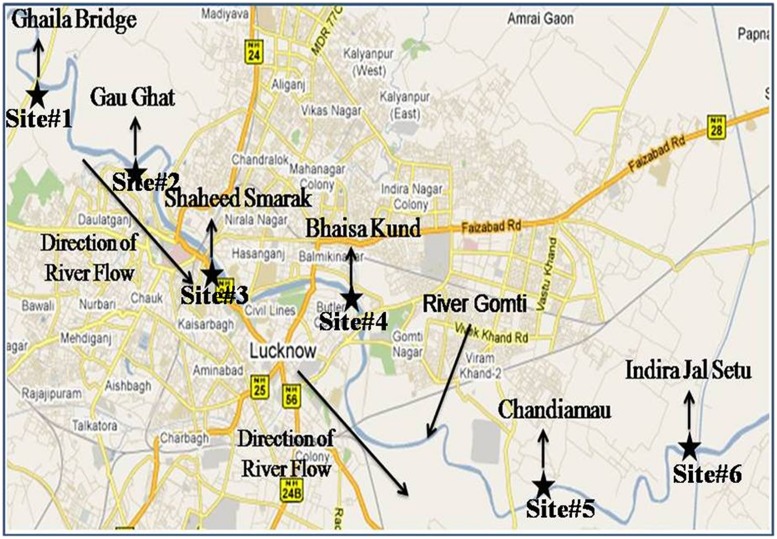
**Sampling sites of surface water, hydrophytes, and sediments in river Gomti in Lucknow city**. The figure was taken and redrawn from Map of India – map data ©2016 Google.

The study was conducted around 20 km stretch of river Gomti in Lucknow city (Latitude: 25.55′ N, Longitude: 80.58′ E and Altitude: 123 m). River Gomti, flowing into the northern part of the country, is a major tributary of the river Ganga in India. Originating from a natural reservoir in the swampy and densely forested area near Madho-Tanda (altitude 200 m; latitude 28°34′ N and E longitude 80°07′ E) in the foothills of Himalayas. The river traverses a distance of about 730 km in the Indo-Gangetic alluvial region before its confluence with river Ganga. The average flow of the river at Lucknow varies between 500 mLd (million liters daily) in summers and 55,000 mLd during the monsoons. The river carries water throughout the year and exhibits sluggish flow except for the monsoon period. For this study, six sampling sites were selected in an up-to-downstream fashion along the river. The sampling sites were Ghaila-Bridge (site 1# located in uppermost stream of river from where river enters the Lucknow city), Gau-Ghat (site 2# Cloth washing and bathing spot), Shaheed Smarak (site 3# bathing ghat and holy spot), Bhaisakund (site 4# human cremation spots), Chandiamau (site 5# Bathing and human activities), and Indira jal-Setu (site 6# down-most stream location in the landscape; **Figure 2**). Samples were collected in triplicate (*n* = 18). In brief, three transects were established randomly at each site and 1 l water samples were collected in sterile glass bottles from 30 cm below the water surface from the left bank, midstream, and right banks and transported on ice to the laboratory for analysis. Sample processing and analysis were conducted within 6 h after sample collection. DNA isolation was carried out by boiling lysis ethanol precipitation method.

**FIGURE 3:**
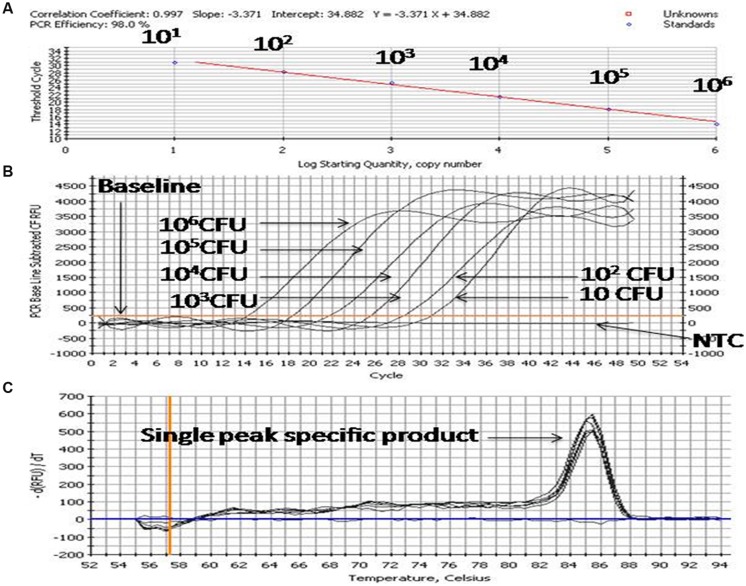
**PCR profiling and melting curve analysis of qPCR **(A–C)**.** **(A)** Standard curve, **(B)** Amplification curve, **(C)** Melt curve. Product (length 154 bp) amplified targeting the 23S rRNA gene of 10-fold serially diluted cultures of *Enterobacter* MTCC 569.

#### Collection of Hydrophytes from Riverine-System and DNA Isolation

The same sampling sites as for surface water (**Figure 2**) were selected and three transects were made randomly to collect hydrophytes. The submerged hydrophytes *Potamogeton crispus* (L.), commonly known as curly-leaf pondweed were taken into consideration. The samples were collected as per APHA protocol (American Public Health Association [APHA], 1998). Along with each transect, 1 m^2^ quadrats were set at a distance of 0.5 m from left and right banks. All the *P. crispus* plants within the quadrats were collected in separate sterile zip-lock bags. Along with hydrophytes, 1 l surrounding waters was also collected in sterilized bottles. Further, plant-associated microbiota was recovered in 500 mL phosphate buffered saline and it was pooled with 500 mL surrounding river water. A 10-fold diluted portion of this sample was used for estimation of CFU/100 mL water. Briefly, *P. crispus* (10 ± 0.5 g fresh weight) were sonicated for 30 s in 350 mL phosphate buffered saline and then kept on a rotary shaker (INNOVA 4230) for 10 min to release the plant associated microbiota. Subsequently plants were rinsed thrice in 50 mL phosphate buffered saline. The sonication (amplitude: 30%, pulse time: 0.5 s.; UP200S Ultrasonic processor, Dr. Hielscher GmbH, Germany) of the plant followed by three sequential rinsing provided an average recovery of 95% of the bacteria from the plant surface. Further, the rinse which mostly contains bacteria was then carefully removed for isolation and purification of multigenomic DNA using boiling lysis followed by precipitation by sodium acetate-ethanol method. The quantity and quality of extracting DNA were measured by NanoDrop spectrophotometer ND 3.0 1000 (NanoDrop Technologies, Wilmington, DE, USA).

#### Collection of Sediments from Riverine System and Isolation of Multigenomic DNA

Same sampling sites as for surface water were selected for the sediment collection in river Gomti (**Figure 2**). Sediment core samples (∼250 g) were collected directly from an approximately 0.5 cm depth of the sediment surface at three different positions (∼1 m distance) of each sampling site from both banks of the river using a sterile stainless steel and placed in plastic bags. All the samples (*n* = 6) from each sampling site were mixed and prepared the grab sample. Final 10 ± 0.5 g fresh weights were taken for DNA isolation. Samples (10 ± 0.5 g) from each site were added to 100 ml of sterile phosphate-buffered saline. After vortexing, the grab samples were allowed to shake in incubator shaker (INNOVA 4230) at 220 rpm at 37°C for 10 min. Further, the samples were allowed to gravity settle at the bottom of the tube. The supernatant which mostly contains bacteria was then carefully removed for isolation and purification of multigenomic DNA using boiling lysis followed by precipitation by sodium acetate-ethanol method. The quantity and quality of extracting DNA were measured by NanoDrop spectrophotometer ND 3.0 1000 (NanoDrop Technologies, Wilmington, DE, USA).

### Statistical Analysis

For comparison of PCR amplification efficiencies and detection sensitivities among different experiments, the slopes of the standard curves were calculated by performing a correlation and regression analysis through iCycler iQ^TM^ Real-Time Detection System Software Version 3.0 A. Amplification efficiency (*E*) was estimated by using the slope of the standard curve and the formula *E* = (10^–1/slope^) – 1. A reaction with theoretical 100% efficiency will generate a slope of –3.322. Data obtained from conventional and qPCR were compared by Wilcoxon Rank-Sum Test. *Enterobacter* sp. retrieved in this study at different sites was analyzed using one way analysis of variance (Gomez and Gomez, 1984).

## Results

The SYBR Green based qPCR assays targeting 16S rRNA, 23S rRNA genes were used to detect *Enterobacter* quantitatively, in the potable water samples collected from urban boundaries of a city Lucknow. A significant variations in, *Enterobacter* contamination were observed at different sites (one-way ANOVA, *p* < 0.05). The *Enterobacter* levels at sites: A–J targeting 16S rRNA, 23S rRNA genes were 13320 ± 428.90 CFU/100 ml, ND (not detected), ND, ND, 1304 ± 34.94, 1290 ± 32.89, 1460 ± 39.42, ND, ND, and ND, respectively (**Table 2**). These findings showed that the *Enterobacter* were present at the sampling site: A (the surface water) and site: E, site: F site: G (the potable water) only.

**Table 2:** *Enterobacter* count in potable water (per 100 ml).

^a^Sampling sites	Sites name	*Enterobacter* (per 100 ml)^b,c^
Site : A	Gaughat	13320 ± 428.90
Site : B	Aishbagh	ND^d^
Site : C	Husain Ganj	ND
Site : D	Kaiserbagh	ND
Site : E	Nakkhas	1304 ± 34.94
Site : F	Shastrinagar	1290 ± 32.89
Site : G	Saadatganj	1460 ± 39.42
Site : H	City Station	ND
Site : I	Motinagar	ND
Site : J	KGMU	ND

^a^*Samlping site: A represent surface (river) water whereas, sites: B–J represent potable water.*

*Site A: raw water intake point in the river Gomti, site B: Aishbagh Waterworks before water enters the distribution system; sites C,D: water-distribution pipeline that neither percolated nor ran along open drainage; sites E–J: pipeline that percolated and ran along open drainage*, ^b^*nuclease-free water used as negative Control*, ^c^*One-way ANOVA, p < 0.05 for each column*, ^d^*ND: not detected.*

Surface water is the most potent source of microorganisms. The bacterial load represents the level of contamination in the river. The *Enterobacter* levels in surface water were 1.76 × 10^4^ ± 492, 1.33 × 10^4^ ± 334, 1.15 × 10^4^ ± 308, 2.56 × 10^4^ ± 802, 2.89 × 10^4^ ± 962, and 8.16 × 10^4^ ± 3443 cfu/100ml at sampling site # 1, Site # 2, Site # 3, Site # 4, Site # 5 and Site # 6 respectively (**Table 3**).

**Table 3:** *Enterobacter* count in river Gomti in surface water (per 100 ml), sediments (per 10 g) and hydrophytes (per 10 g).

Sampling sites	Sites Name	Surface water	Sediments	Hydrophytes
Site # 1	GhailaBridge	1.76 × 10^4^ ± 492	2.36 × 10^4^ ± 703	4.80 × 10^4^ ± 1804
Site # 2	Gaughat	1.33 × 10^4^± 334	1.98 × 10^4^ ± 530	3.48 × 10^4^± 856
Site # 3	Shaheed Smarak	1.15 × 10^4^± 307	9.92 × 10^4^ ± 3839	8.50 × 10^4^± 2074
Site # 4	Bhaisakund	2.56 × 10^4^± 801	6.80 × 10^4^± 2230	8.09 × 10^4^ ± 1724
Site # 5	Chandiamau	2.89 × 10^4^± 962	8.76 × 10^4^± 3066	6.30 × 10^4^ ± 1738
Site # 6	Indira Jal Setu	8.16 × 10^4^ ± 3443	2.34 × 10^4^ ± 732	3.68 × 10^4^ ± 949

Hydrophytes become the stationary/static habitats of bacteria in river water. Bacteria get attached to parts of the plants for example leaves, stems or on the roots of the floating plants. The levels of *Enterobacter* contamination associated with hydrophytes were 4.80 × 10^4^ ± 1804, 3.48 × 10^4^ ± 856, 8.50 × 10^4^ ± 2074, 8.09 × 10^4^ ± 1724, 6.30 × 10^4^ ± 1738 and 3.68 × 10^4^ ± 949 cfu/10 g at sampling Site # 1, Site # 2, Site # 3, Site # 4, Site # 5 and Site # 6 respectively (**Table 3**).

The sediments are the silent reservoir of microorganisms in the rivers. By analyzing the sediments the inflow of drainage into the river system can be predicted. Sediments can also be one of the important factors which can be helpful in microbial source tracking. The *Enterobacter* found in sediments were in the range of 2.36 × 10^4^ ± 703, 1.98 × 10^4^ ± 530, 9.92 × 10^4^ ± 3839, 6.80 × 10^4^ ± 2230, 8.76 × 10^4^ ± 3066 and 2.34 × 10^4^ ± 732 cfu/10 g at sampling Site # 1, Site # 2, Site # 3, Site # 4, Site # 5, and Site # 6, respectively (**Table 3**).

## Discussion

The detection limit of the qPCR assay was 10 CFU/100 ml of water. In this study, the occurrence of *Enterobacter* in different environmental samples delineates their impact on the environment. The *Enterobacters* were present in potable water at sampling site: E, site: F, and set: G only. The localized occurrence of *Enterobacter* was due to local pollution. The *Enterobacter* loads at these sites were more than 1000 CFU/100 ml and this condition may be treated as the alarming situation for human health.

Musgrove et al. (2008) reported that the members of family Enterobacteriaceae namely *Escherichia, Enterobacter, Citrobacter*, and *Kiebsielia* were the most commonly encountered genera in shell egg processing plants. The eﬄuents from various kinds of food processing industries may lead to the discharge of its waste materials into riverine systems. Some of the microorganisms get settled down into the sediments, some get adhered to hydrophytes, while the remaining gets suspended in water bodies.

**FIGURE 4:**
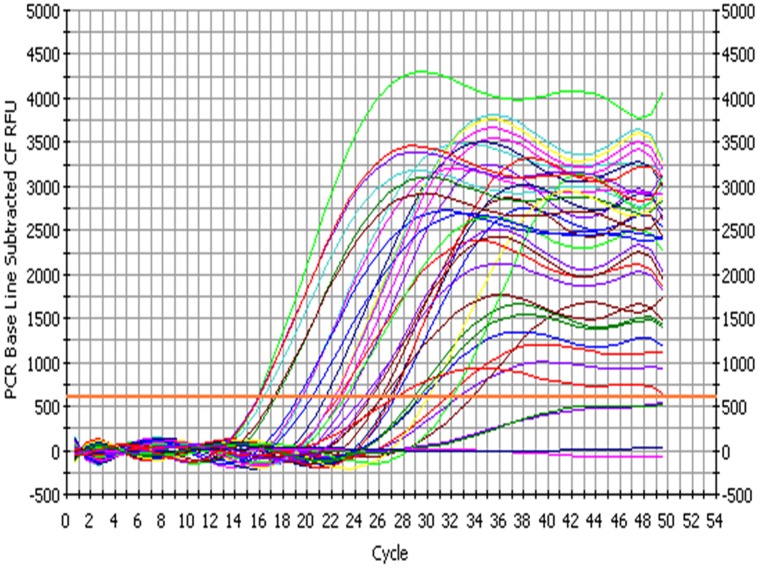
**Amplification profile of 23S rRNA gene of *Enterobacter* for culture-independent detection of *Enterobacter* in environmental samples**.

**FIGURE 5:**
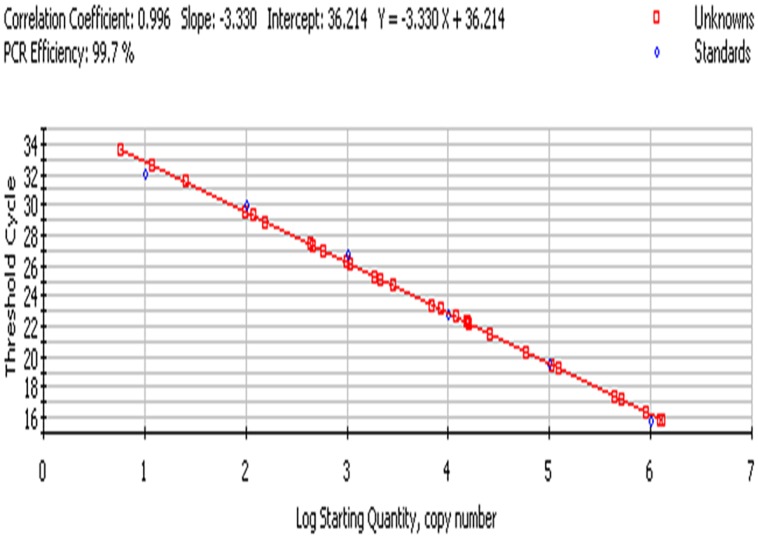
**Standard curve; culture-independent quantification of *Enterobacter* targeting 23S rRNA gene in environmental samples**.

The bacterial load on the riverine system can be identified in three domains of the river that are (1) in surface water, (2) associated with plants in rivers and (3) in the sediments of the river. In this study, we analyzed all the three domains to identify the bacterial load to study the ecological impact of *Enterobacter* and vice versa (**Figure 4** and **Figure 5**).

**FIGURE 6:**
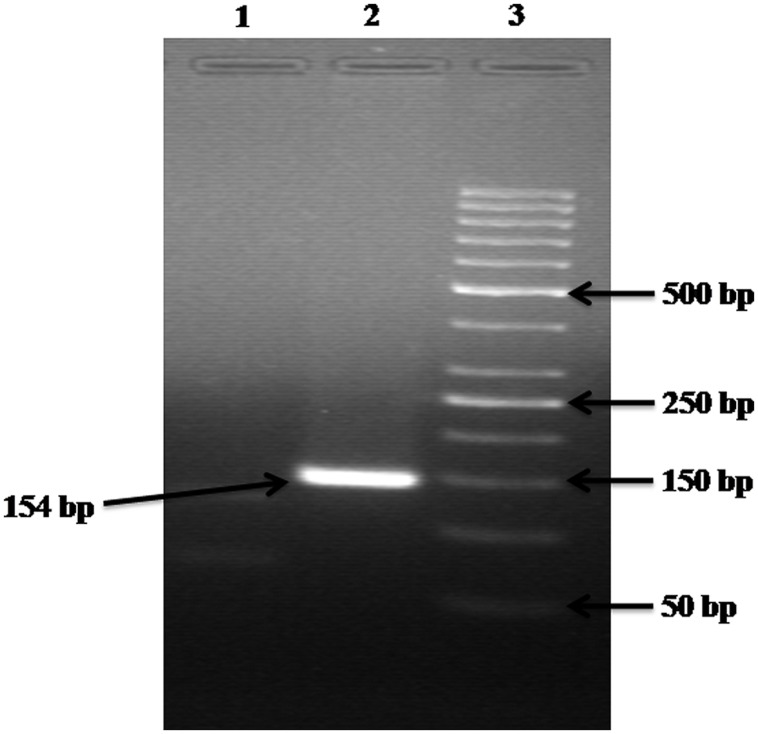
**Gel electrophoresis analysis of control experiments**. Lane: 1 contained the DNA of *Escherichia coli, Citrobacter*, and *Klebsiella* but NOT of *Enterobacter*; hence no product is formed. Lane: 2 contained the DNA of *Escherichia coli, Citrobacter, Klebsiella*, and *Enterobacter*; hence a 154 bp product is formed. Lane: 3 carry 50 bp ladder.

The natural water reservoirs have self-sustenance capability to manage and keep ecological balance through various mechanisms, including solar inactivation of pathogens by UV-B solar radiation and interaction of sunlight generated reactive oxygen species (ROS) with external sensitizer molecules (Beuchat, 2002; Kohn and Nelson, 2007). In the ecosystem of the river the survival rates and die-offs of these biological contaminants are also governed through grazing and predation at various trophic levels (Peters, 1994; Sherr and Sherr, 2002; Sinton et al., 2007). However, the uninterrupted anthropogenic waste disposal in the small tributary rivers has overburdened these natural resources leading to waterborne diseases and outbreaks. The sources of pollution are more responsible for the declining of quality of water. The detection of fecal Coliforms in river waters, sediments of the river and aquatic plant associated microbiota is important not only because this group has been used as an indicator of microbial quality of water but also due to their role in the dissemination and persistence of antimicrobial resistance (Sletvold et al., 2007). Earlier investigation on persistence of fecal indicator bacteria *Escherichia coli* and enterococci in mats of the green alga *Cladophora glomerata* (L.) kütz have concluded that such associations serve as an environmental source of indicator bacteria (Whitman et al., 2003). Additionally, the discharge of untreated sewage and waste-water into the river might be a contributing factor in the prevalence of antimicrobial-resistant microorganisms.

According to Dufour (1977) and Leclerc et al. (2001), the ratio of *Escherichia coli:Citrobacter*/*Enterobacter:Klebsiella* were, 94:4:2 in natural habitats. But in this study, we have observed significant variations in their relative ratios. In surface water of river Gomti the contamination levels of fecal coliforms were two to five higher orders of magnitude (**Table 3**) and the pattern of contamination were in increasing orders from upstream to downstream fashion. The fecal coliforms ratio in surface water (*n* = 36) from six sampling sites were, 88.89:4.78:5.11:1.52 of *Escherichia coli*:*Enterobacter*:*Citrobacter*:*Klebsiella*, respectively (data not shown).

Aquatic flora may be an important and significant non-point source of bacteria in water reservoirs (Scott et al., 2002). Aquatic macrophytes form strands in polluted eutrophicated water bodies impacted by urban sewage. There are several reports which demonstrate the occurrence and growth of indicator bacteria in aquatic hydrophytes (Byappanahalli et al., 2003; Whitman et al., 2003; Ishii et al., 2006). Further, bacteria adhered on plants may detach from their natural strands naturally or by anthropogenic activities, could float several miles downstream along the course of the river, and get transported to distant destinations. Diarrheal disease caused by ETEC (Enterotoxigenlic *Escherichia coli*) and other diseases caused by fecal coliforms is the main cause of death in infants and small children in developing countries (Begum et al., 2005, 2007). Despite the potential public health threat from water- and foodborne ETEC, regulatory authorities in the developing world rely exclusively on “indicators” of fecal pollution (e.g., fecal coliform bacteria or generic *Escherichia coli*) for determination of water quality. This fails to provide a clue on presence of pathogenic forms of *Escherichia coli*, viz., ETEC present in the contaminated source. Therefore, the need for surveillance of water reservoirs and potential non-point sources for *Enterobacter* and pathogenic *Escherichia coli* including ETEC in the developing world has been realized. In river Gomti due to its sluggish flow, the fecal coliforms levels were four to six higher orders of magnitude and the bacterial concentrations were in increasing order from upstream to downstream fashion with exceptions (**Table 3**). This delineates the relative abundance of *Enterobacter* at each sampling site and the data shows that there is no fixed pattern and this may be due to unequal local distribution of the nutrients and point as well as non-point sources of pollution. The analysis of (*n* = 36) plant samples in depicting that the percent ratio of fecal coliforms were 83.44:10.51:3.64:2.42 of *Escherichia coli*:*Enterobacter*:*Citrobacter*:*Klebsiella* were, respectively.

Sediments are rich in the microbial diversity, since large numbers of microorganisms are attached to suspended particles and sediments (Fish and Pettibone, 1995; Crump and Baross, 1996). In areas adjacent to the rivers, the chances of risk of infection to human population are high as the potentially pathogenic microorganisms from the sediment layer are re-suspended during recreational activities. Along with sediments, most of the pollutant which cannot dissolve and the biological component such as microorganisms also get settled down and hence, the sediments become the silent reservoir of microbes in riverine environments. The concentration of *Enterobacter* counts was four to five higher orders of magnitude (**Table 3**). By analyzing the sediment samples (*n* = 36) from six sampling sites the relative percentage fecal coliforms ratio was 60.22:7.39:26.69:5.74 of *Escherichia coli*:*Enterobacter*:*Citrobacter*:*Klebsiella*, respectively. In general, at sampling site # 1 (upstream) the levels of all the fecal coliforms were minimum, whereas at sampling site # 6 (down-most streams) the bacterial levels were maximum and this progression were in increasing order with few exceptions.

The bottom of river Gomti is mostly marshy due to its sluggish flow, which leads to the depletion of oxygen and hence high biochemical oxygen demand (BOD; Singh et al., 2004). High BOD may lead to change in the ecosystem of the bottom of the river and hence the unusual distribution of microbiota will be possible. The major conclusion of the study is that this method is rapid and sensitive enough to detect *Enterobacter* species from water samples. And this method could be used in the monitoring of water contamination with *Enterobacter*.

## Author Contributions

CP: Sample collected, executed the experiments, and designed the manuscript. RS: Designed the experiments and strategy of the sampling. VG: Resolution of the critical questions related to the accuracy of data. RU: Analyzed as well as interpreted the data.

## Conflict of Interest Statement

The authors declare that the research was conducted in the absence of any commercial or financial relationships that could be construed as a potential conflict of interest.

## Footnotes

1 http://www.ncbi.nlm.nih.gov

2 www.ebi.ac.uk/clustalW

3 http://www.ncbi.nlm.nih.gov/BLAST/

4 http://ehp03.niehs.nih.gov/article/fetchArticle.action?articleURI=info:doi/10.1289/ehp.10809
